# Supplementary material for: The causal role of circulating inflammatory markers in osteoporosis: a bidirectional Mendelian randomized study
Source: Front Immunol. 2024 Jul 18;15:1412298. doi: 10.3389/fimmu.2024.1412298 (PMC11291241; doi:10.3389/fimmu.2024.1412298)
Supplement: Supplementary file 1 [file DataSheet_1.zip › supplementary material/CIM-OSTEOPOROSIS /GCST90274796.csv_forest.pdf]

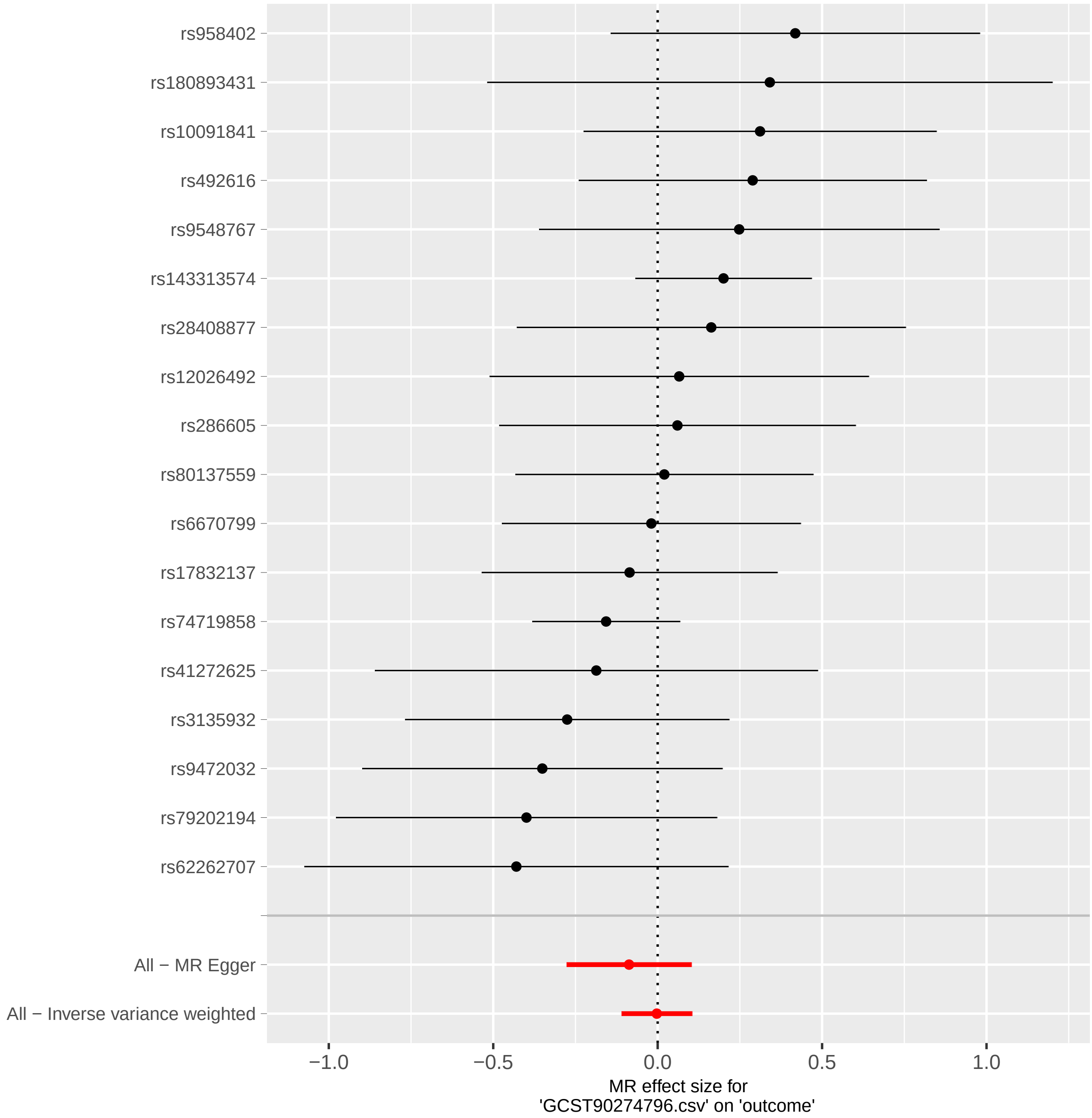
rs958402
rs180893431
rs10091841
rs492616
rs9548767
rs143313574
rs28408877
rs12026492
rs286605
rs80137559
rs6670799
rs17832137
rs74719858
rs41272625
rs3135932
rs9472032
rs79202194
rs62262707
All − MR Egger
All − Inverse variance weighted
−1.0
−0.5
0.0
0.5
1.0
MR effect size for
'GCST90274796.csv' on 'outcome'
